# Endocytotic Uptake of Zoledronic Acid by Tubular Cells May Explain Its Renal Effects in Cancer Patients Receiving High Doses of the Compound

**DOI:** 10.1371/journal.pone.0121861

**Published:** 2015-03-10

**Authors:** Anja Verhulst, Shuting Sun, Charles E. McKenna, Patrick C. D’Haese

**Affiliations:** 1 Laboratory of Pathophysiology, University of Antwerp, Antwerp, Belgium; 2 Department of Chemistry, University of Southern California, Los Angeles, California, United States of America; Anatomy, SWITZERLAND

## Abstract

Zoledronic acid, a highly potent nitrogen-containing bisphosphonate used for the treatment of pathological bone loss, is excreted unmetabolized via the kidney if not bound to the bone. In cancer patients receiving high doses of the compound renal excretion may be associated with acute tubular necrosis. The question of how zoledronic acid is internalized by renal tubular cells has not been answered until now. In the current work, using a primary human tubular cell culture system, the pathway of cellular uptake of zoledronic acid (fluorescently/radiolabeled) and its cytotoxicity were investigated. Previous studies in our laboratory have shown that this primary cell culture model consistently mimics the physiological characteristics of molecular uptake/transport of the epithelium *in vivo*. Zoledronic acid was found to be taken up by tubular cells via fluid-phase-endocytosis (from apical and basolateral side) as evidenced by its co-localization with dextran. Cellular uptake and the resulting intracellular level was twice as high from the apical side compared to the basolateral side. Furthermore, the intracellular zoledronic acid level was found to be dependent on the administered concentration and not saturable. Cytotoxic effects however, were only seen at higher administration doses and/or after longer incubation times. Although zoledronic acid is taken up by tubular cells, no net tubular transport could be measured. It is concluded that fluid-phase-endocytosis of zoledronic acid and cellular accumulation at high doses may be responsible for the acute tubular necrosis observed in some cancer patients receiving high doses of the compound.

## Introduction

Zoledronic acid or zoledronate (Zometa; Novartis Pharma AG, Basel, Switzerland) is a third generation, highly potent nitrogen (N)-containing bisphosphonate that has shown beneficial effects in the treatment of hypercalcemia of malignancy and skeletal metastases in patients with multiple myeloma, lung, breast, and prostate cancer [1–3].

N-containing bisphosphonates, including zoledronic acid, all show a high affinity for the bone mineral hydroxyapatite (HAP). Therefore these drugs, when circulating in the blood compartment, rapidly bind to bone tissue, (±60% of the administered dose of zoledronic acid is retained in the bone) [4] and are presumably released from it during the process of bone resorption. Once adsorbed into the bone mineral, N-containing bisphosphonates are internalized via fluid phase endocytosis by the osteoclast where they are believed to inhibit farnesyl diphosphate synthase, the isoprenoid biosynthetic enzyme in the cholesterol biosynthesis pathway [5]. Disruption of a branch pathway of the cholesterol biosynthesis pathway i.e. isoprenylation then results in the pharmacological activity of N-containing bisphosphonates [6]. Isoprenylation involves covalent linkage of farnesyl diphosphate or geranylgeranyl diphosphate to the carboxy-terminus of regulatory proteins, including the small GTPases Ras, Rac, Rho and Cdc42. The latter three, as well as numerous others, are geranylgeranylated and play a rate-limiting role in the resorptive activity of osteoclasts [7–9].

Zoledronic acid not bound to the mineralized bone is excreted, unmetabolized by the kidney, predominantly within the first hours after administration [4;10]. Cancer patients frequently receive a monthly dose of 4 mg of zoledronic acid infused intravenously in 100ml fluid over 15 minutes. In some of these patients renal excretion of high circulating amounts may be associated with acute tubular necrosis, characterized by tubular cell degeneration, loss of brush border, and apoptosis [11]. A relationship exists between peak levels of zoledronic acid in the blood and renal toxicity since renal damage declines when the dose is reduced or the infusion time is extended [1]. Moreover, the fact that renal toxicity is not observed in postmenopausal women receiving only an annual dose of 5 mg zoledronic during treatment of osteoporosis is in line herewith [12].

It is not yet understood by which pathway zoledronic acid is taken up by the renal tubular cells. On the other hand, it is known that the degree by which zoledronic acid and other N-containing bisphosphonates are taken up by different cell types is proportional to their capacity for fluid phase endocytosis [13–15]. Furthermore, it is also possible that zoledronic acid uses pathways of transcellular organic anion transport involved in the renal excretion of many other drugs [16–18].

A cell culture system of primary human tubular kidney cells has been developed in our laboratory. The in vitro model was characterized extensively both at the physiological and pathophysiological level and evidence was presented for these cultures to consistently mimic the most important physiological characteristics of molecular uptake/transport by the tubular epithelium *in vivo* [19–23]. As we previously described, these cultures show both fluid-phase and receptor- mediated endocytotic uptake of molecules [22;24]. In addition they possess the full capacity of controlled transport of molecules, by both anionic and cationic transporter molecules across the epithelium [18] as they express a wide palette of transporters at the mRNA and protein level. At the functional level, the primary human tubular cell monolayers retain the necessary machinery to mediate the net secretion of the prototypic substrates i.e. the organic cation, para-amino hippuric acid (PAH), and the organic anion creatinine.

In order to better understand the observed renal toxicity of zoledronic acid, the aim of the present study was to investigate possible uptake routes and pharmacological handling of zoledronic acid by tubular epithelial cells using the above described primary human cell culture model.

## Materials and Methods

### Primary human tubular kidney cell cultures

Human tubular epithelial cells were isolated from normal human kidney tissue that became available through nephrectomy performed on oncological indication. The use of this tissue for the purpose of cell culture was approved (P2013/268) by the ethical committee of the Erasme Hospital (Brussels, Belgium) involved in tissue collection. Written informed consent was obtained. Macroscopically normal tissue was collected and processed in a sterile manner. Cortex and outer stripe of outer medulla were dissected, decapsulated and cut into pieces of about 1 mm^3^. Afterwards the tissue fragments were digested in collagenase D solution (Roche, Ottweiler, Germany) during 2h at 37°C, under vigorous shaking, and sieved through a 120μm sieve. The resulting cell suspension was loaded on top of a discontinuous Percoll (GE Healthcare, Diegem, Belgium) gradient with densities of 1.04 and 1.07 g/ml. After centrifugation (25min, 1620 rcf), cells from the intersection were carefully aspirated, washed and brought into culture as a mixed population of proximal tubular, distal tubular and collecting duct cells. Tubular cells were grown until confluence (10 to 14 days) on permeable, polycarbonate filter supports (Costar, Corning, NY, USA) at a density of 50.000 cells/filter, in a-MEM (Life Technologies, Gent, Belgium) modified according to Gibson d’Ambrosio [25] supplemented with 10% fetal calf serum (FCS). Cell cultures grown on 6.5mm permeable (0.4μm pore size) filter supports (Costar) are allowed to polarise and have a separated apical and basolateral compartment.

In order to avoid the use of leaky (non-confluent) cultures, confluence of the cell cultures was assessed by measuring the transepithelial resistance (TER) of the monolayers. Monolayers were not used for further experiments if the transepithelial resistance of the monolayer, corrected for the resistance of the filter, was less than 55 Ω.cm^2^ (>2xSD below the mean TER at the start of the experiments). TER was measured using an epithelial voltohmmeter equipped with a STX2 electrode (World Precision Instruments, Hitchin, UK). FCS-containing medium was replaced by serum-free medium or Krebs solution prior to the experiments described in the following paragraphs

### Measurements of cell viability/monolayer integrity

TER was measured before and after a 2h incubation period with different zoledronic acid concentrations (0, 0.1, 1, 10 and 100 μM). Cell viability was then evaluated using an MTT based assay (Easy for you, Biomedica Gruppe, Vienna, Austria), according to the manufacturer’s instructions. Cell viability was also recorded 4, 24 and 48h after the 2h incubation period with various zoledronic acid doses (0, 1, 5 and 100μM) and following 4, 24 and 48h incubation with zoledronic acid (0, 1, 5 and 100μM). Experiments measuring cell viability/monolayer integrity were performed on monolayers originating from 4 different kidney specimens. For each experiment at least 4 monolayers/condition were used.

### Uptake of fluorescently labeled zoledronic acid in primary human tubular kidney cell cultures

Zoledronic acid was fluorescently labeled with 5-carboxyfluorescein [5-FAM] or Alexa Fluor 647 [AF647]. 5-FAM and AF647 were obtained from Invitrogen. Fluorescent labeling was performed by stable conjugation of the succinimidyl ester of the fluorophore to the imidazole nitrogen of zoledronic acid via the linker strategy, as described previously by McKenna [26;27]. The labeled zoledronic acid was purified by HPLC and characterized by UV, fluorescence, ^1^H and ^31^P NMR and by MS as described previously [26;27] and subsequently dissolved in PBS.

Confluent monolayers of primary human tubular kidney cells were incubated either at the apical or basolateral side with 5-FAM/AF647 labeled zoledronic acid (50μM) for 1 hour at either 37 or 4°C. Monolayers were then formalin fixed and counterstained with Hoechst 33258. This experiment was performed on monolayers originating from two different kidney specimens.

In order to check for the fact that the zoledronic acid signal indeed was localized inside the cells, the cell membrane of proximal tubular cells was labeled following incubation with zoledronic acid and fomalin fixation. For this purpose the monolayers were incubated for 20 min with normal donkey serum (20%), and for 2 hours with a mouse monoclonal antibody to human leucine aminopeptidase, a specific proximal tubular cell marker [19;28;29]. Subsequently an AF488-labeled donkey anti mouse secondary antibody (Life technologies, Gent, Belgium) was used and monolayers were counterstained with Hoechst 33258.

Fluorescent signals were evaluated using confocal microscopy (Perkin Elmer, Zaventem, Belgium) and application of Volocity software (Perkin Elmer).

### Identification of zoledronic acid-containing intracellular vesicles

In order to investigate whether the cellular uptake of zoledronic acid took place by fluid phase endocytosis and/or receptor mediated endocytosis, confluent monolayers were co-incubated with AF647-labeled zoledronic acid (50μM) and FITC-labeled dextran or FITC-labeled albumin, at either the apical or basolateral side at 37°C for 1 hour. FITC- labeled dextran and FITC-labeled albumin are established markers of fluid phase and receptor-mediated endocytosis, respectively [14;30;31]. This experiment was performed on monolayers originating from two different kidney specimens.

Furthermore, confluent monolayers of human tubular kidney cells were incubated with cytochalasin B (45 min, 30μM) before exposing cells to AF647-labeled zoledronic acid, or FITC-labeled dextran or FITC-labeled albumin. Cytochalasin B, a cell-permeable mycotoxin that strongly inhibits network formation by actin filaments, in general is recognized as an inhibitor of endocytosis. However, cytochalasin B does not affect [32–34] the uptake of FITC-dextran by fluid phase endocytosis.

Fluorescent signals were evaluated using confocal microscopy (Perkin Elmer, Zaventem, Belgium) and application of Volocity software (Perkin Elmer).

### Measurement of intracellular levels of radiolabeled zoledronic acid

The intracellular concentration of zoledronic acid was quantified after creating steady state conditions. Steady state conditions were obtained as described previously (Simmons, 1990), by adding the same concentrations (5μM) of zoledronic acid in pre-warmed Krebs buffer to both the apical and basolateral side of the polarized cell cultures. After 1 hour of incubation, ^14^C labeled zoledronic acid (specific activity 2.04 GBq/mmol, provided by Novartis Pharma AG, Basel) was added at a concentration of 1μCi/ml to the cell culture medium, at either the apical or basolateral side of the monolayer. After an additional incubation period of 1 hour the permeable supports were cut out and the number of disintegrations per minute was measured in a scintillation counter. Intracellular levels of zoledronic acid are presented as pmoles/cm^2^. The experiment was performed 4 times in monolayers originating from 4 different kidney specimens. For each experiment at least 5 monolayers/condition were used.

### Comparison of intracellular radiolabeled zoledronic levels acid to intracellular radiolabeled mannitol levels

On monolayers of 1 kidney (5 monolayers/condition), intracellular levels of zoledronic acid were compared to those of mannitol, a molecule which is not internalized by tubular cells [18;35]. Cellular accumulation of mannitol was investigated by performing an experiment identical to that described for zoledronic acid on parallel monolayers of the same kidney. ^3^H-labelled mannitol was obtained from Perkin Elmer (specific activity 432,9 GBq/mmol).

### The effect of an excess of para-ammino hippuric acid, estrone-3-sulphate or pamidronate on intracellular levels of radiolabeled zoledronic acid

The effect of the organic anion transporter substrates (PAH and estrone-3-sulphate (E-3S), and an additional N-containing bisphosphonate (pamidronate) on cellular uptake of zoledronic acid was investigated by co-incubation of the ^14^C labeled-zoledronic acid with an excess amount of these molecules (50 μM) as described earlier [18;35]. The experiment was performed in monolayers originating from 2 different kidney specimens with at least 5 monolayers/condition.

### Measurement of transepithelial transport of radiolabeled zoledronic acid and comparison to the transepithelial transport of radiolabeled mannitol

In order to measure the transport of zoledronic acid through monolayers of human tubular epithelial cells a steady state condition was created by incubating the monolayers for 1h with 5μM of the molecule in pre-warmed Krebs buffer at both the apical and basolateral side. Apical to basolateral and basolateral to apical fluxes were then measured in different monolayers. Therefore the ^14^C-labeled molecule was added to either the apical or basolateral side of the cultures. A sample was then taken at the opposite side after 1 hour of incubation (unless mentioned otherwise). The experiment was performed 4 times in monolayers originating from 4 different kidney specimens. For each experiment at least 5 monolayers/condition were used.

Transport of zoledronic acid was compared to that of mannitol in monolayers originating from 2 kidneys (for each experiment at least 5 monolayers/condition). Apical to basolateral and basolateral to apical mannitol fluxes were measured by performing an experiment identical to that described for zoledronic acid on parallel monolayers of the same kidney. ^3^H-mannitol was from Perkin Elmer (specific activity 432,9 GBq/mmol).

### Effect of administered concentration on the intracellular levels and the transepithelial transport of zoledronic acid

The effect of the administered zoledronic acid concentration on intracellular levels and transepithelial transport of the compound was investigated using the steady state set-up as described in the respective paragraphs above. Zoledronic acid was administrated in the following concentrations 0.25, 1, 5, 25 and 100μM. This experiment was performed twice on monolayers originiating from 2 different kidney specimens and for each experiment at least 5 monolayers/condition).

### Statistics

Statistical analysis was performed using IBM SPSS statistics 20. The data were analysed using non-parametric statistics (Mann-Whitney U test) with Bonferroni-correction for multiple comparisons.

## Results

### Epithelial integrity and cellular viability

**Fig 1:**
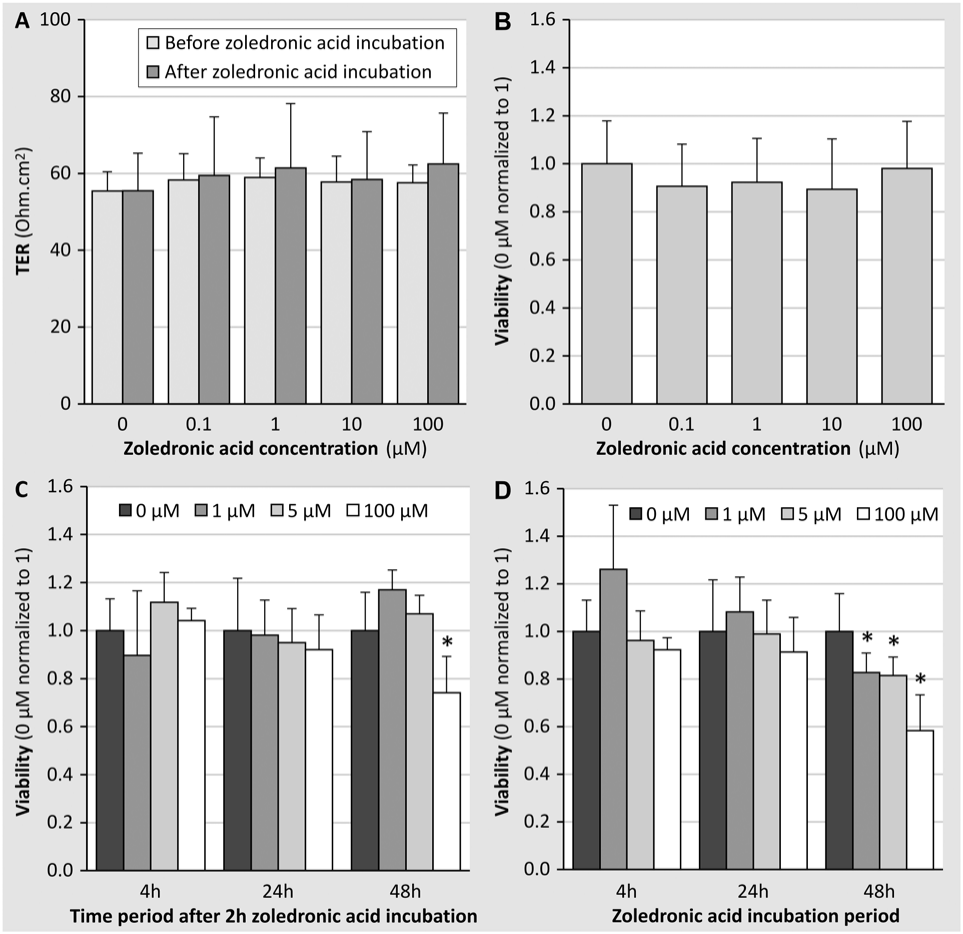
Effect of zoledronic acid incubation on epithelial integrity (evaluated by measuring TER). (A) and viability (evaluated by measuring MTT production) (B) of confluent monolayers of primary human tubular cells. TER was measured before and after a 2 hour incubation period with zoledronic acid (0 to 100μM), after which viability was measured. Each data point represents the mean value ± SD of 4 experiments, performed on cell cultures originating from 4 different kidney specimens. During each of the four experiments at least 4 monolayers/conditions were used. **(C and D)** Effect of zoledronic acid incubation on the viability of confluent monolayers of primary human tubular cells on the longer term. Confluent monolayers of primary human tubular cells were incubated with different (0 to 100μM) concentrations of zoledronic acid for 2 hours and cellular viability was measured 4, 24 and 48 hours later **(C)**. Confluent monolayers of primary human tubular cells were incubated with different concentrations of zoledronic acid for 4, 24 or 48 hours after which cellular viability was measured **(D)**. Each data point represents the mean value ± SD of 2 experiments, performed on cell cultures originating from 2 different kidney specimens. During each of the 2 experiments at least 4 monolayers/condition were used. *p<0.05 vs 0 μM.

Incubation of the monolayers for 2 hours with different concentrations of zoledronic acid (0.1 to 100 μM) did not have a direct effect on epithelial integrity (Fig. 1A) or cellular viability (Fig. 1B). Maintenance of epithelial integrity is critical to study transcellular transport of particular compounds as with too leaky monolayers one would not be able to quantify the transcellular transport as it would be masked by the much higher paracellular transport.

Measurement of cellular viability at 4h, 24h and 48h following a 2 h incubation with different concentrations of zoledronic acid (0 to 100 μM), revealed a significantly reduced viability for the highest zoledronic acid concentration (100μM), 48h post-incubation (p<0.0005) (Fig. 1C). When cells were incubated for 4h, 24h or 48h with the same concentrations of zoledronic acid, a significant, dose-dependent reduction in viability was seen after 48h (Fig. 1D).

### Cellular uptake of zoledronic acid

**Fig 2:**
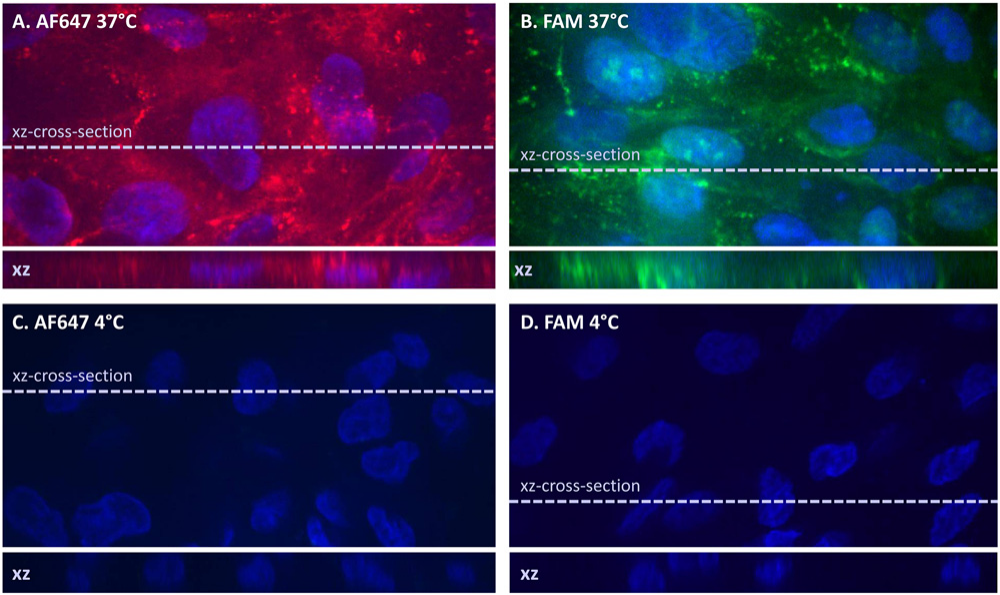
Confluent monolayers of primary human tubular kidney cells were incubated for 1 hour with AF647 (A and C) or FAM (B and D) labeled zoledronic acid (50μM) formalin fixed and counterstained with Hoechst resulting in blue stained cellular nuclei. Monolayer A and B were incubated at 37°C, Cand D at 4°C.

**Fig 3:**
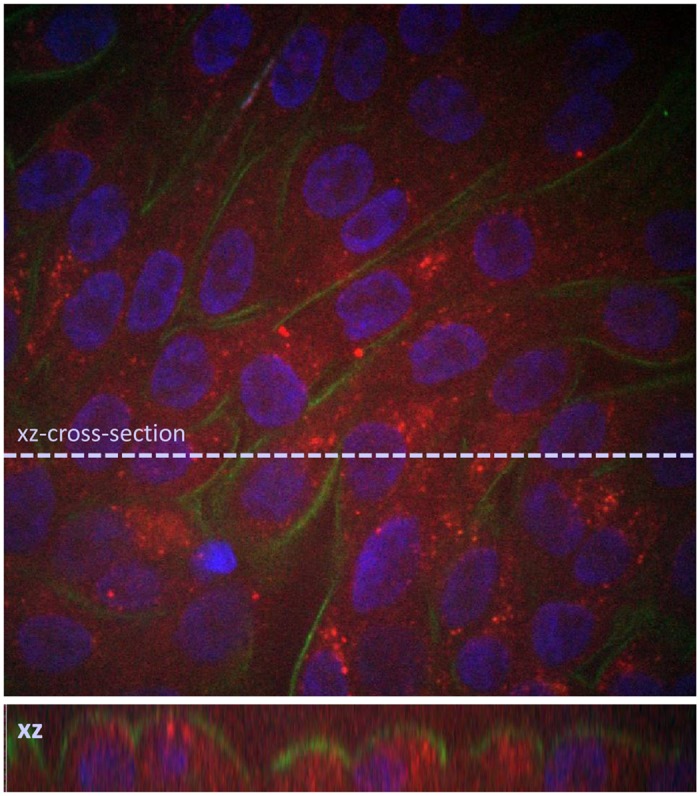
A confluent monolayer of primary human tubular kidney cells was incubated for 1 hour with AF 647- labeled zoledronic acid (50μM) at 37°C (red signal), formalin fixed, immunostained for the proximal tubular membrane marker leucine aminopeptidase (LAP, green signal) and counterstained with Hoechst.

Incubation of primary human tubular monolayers at 37°C with fluorescently (either 5(6)-FAM or AF647) labeled zoledronic acid at the apical membrane resulted in a distinct vesicular fluorescent signal inside the cells (Fig. 2). The intracellular localization of the compound was further evidenced by co-immunostaining of the proximal tubular cell membrane with leucine aminopeptidase (Fig. 3). No difference could be observed in the localization of the fluorescent signal when the fluorescently labeled zoledronic acid was administered at the basolateral instead of the apical side of the monolayers. Furthermore, no signal could be observed when the cells were incubated at 4°C (Fig. 2C-D).

**Fig 4:**
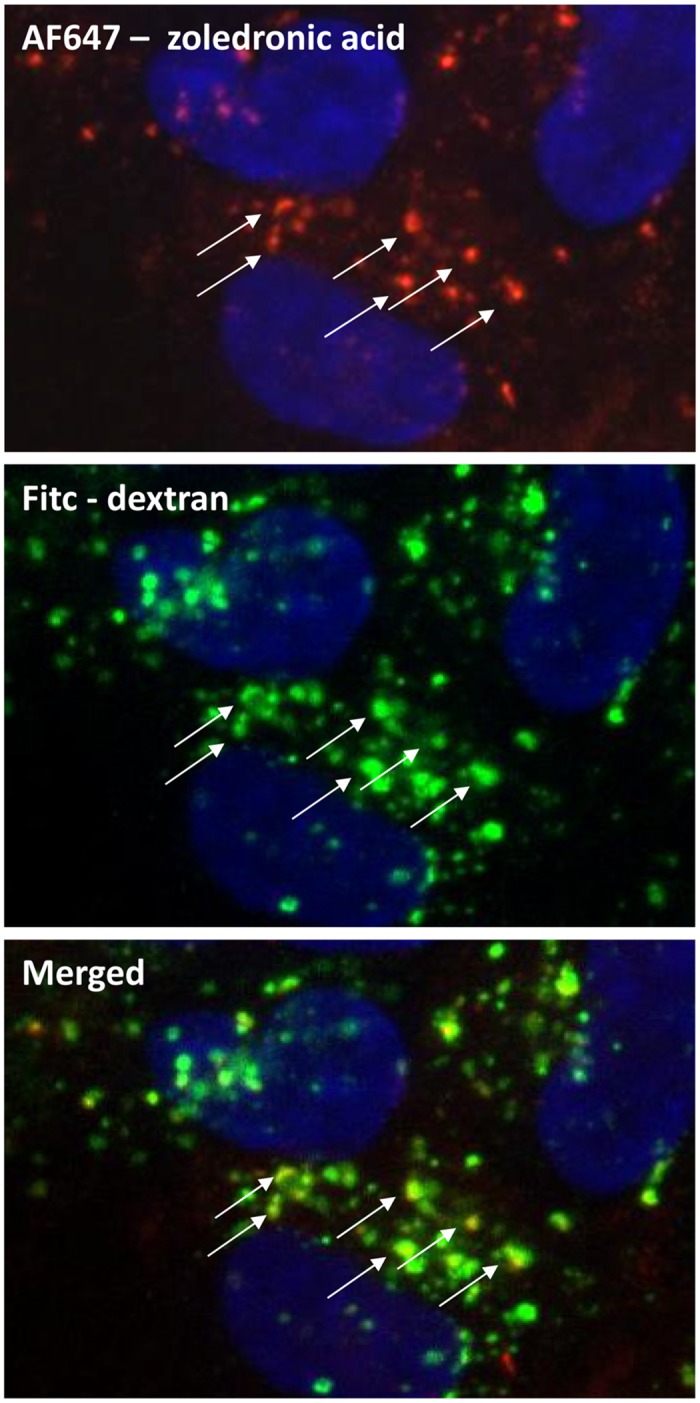
A confluent monolayer of human tubular kidney cells was incubated for 1 hour with AF647-labeled zoledronic acid (50μM) and FITC labeled dextran at 37°C, formalin fixed and counterstained with Hoechst. Arrows clearly show the co-localization of zoledronic acid (red signal) and dextran (green signal) in the same vesicles, staining yellow in the merged figure.

**Fig 5:**
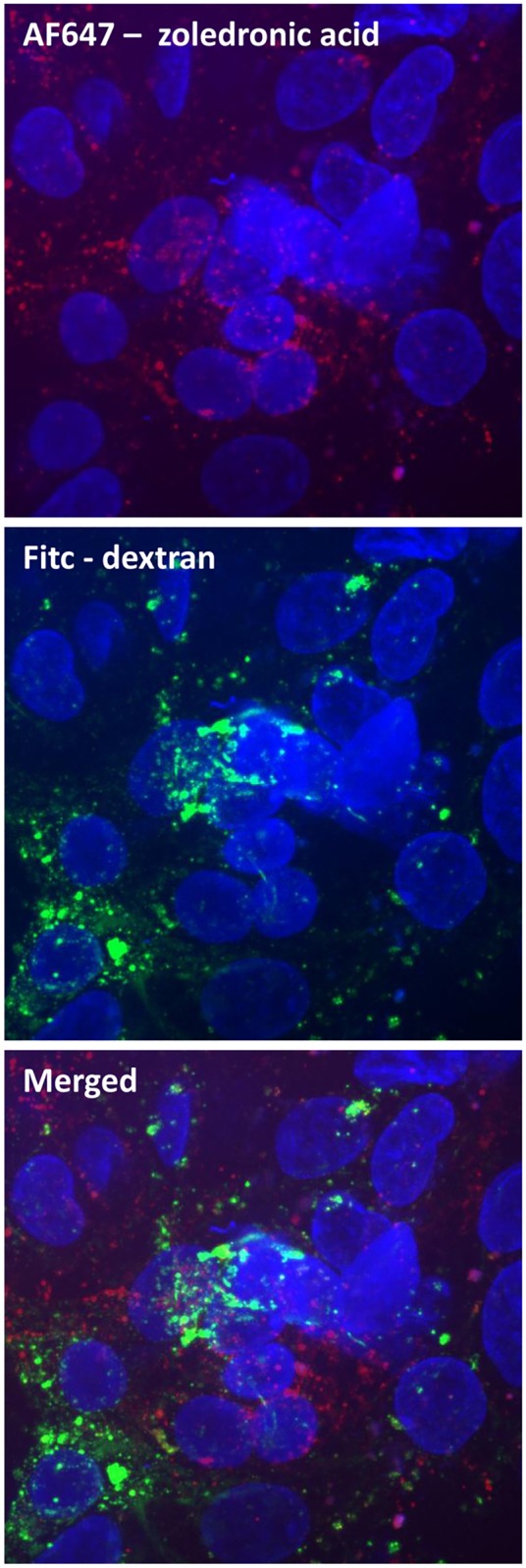
A confluent monolayer of human tubular kidney cells was incubated for 1 hour with AF647-labeled zoledronic acid (50μM) and FITC labeled albumin at 37°C, formalin fixed and counterstained with Hoechst. Zoledronic acid (red signal) and albumin (green signal) clearly are not colocalized.

In order to investigate by which pathway zoledronic acid is taken up into the cells, cellular monolayers were co-incubated with either FITC labeled dextran (established marker of fluid phase endocytosis or FITC labeled albumin (established marker of receptor mediated endocytosis) and AF647 labeled zoledronic acid administered at the apical or basolateral side. As shown on Fig. 4 (uptake from apical side), zoledronic acid and dextran are clearly co-localized in the intracellular vesicles evidencing cellular uptake by fluid phase endocytosis. A similar picture was seen after co-incubation of fluorescently labeled zoledronic acid and dextran at the basolateral side. As shown on Fig. 5 (uptake from apical side), zoledronic acid containing vesicles do not co-localize with vesicles containing albumin, implicating that zoledronic acid is not taken up by receptor-mediated endocytosis which is further evidenced by the fact that, in contrast to zoledronic acid, albumin is almost not taken up from the basolateral side (data not shown).

**Fig 6:**
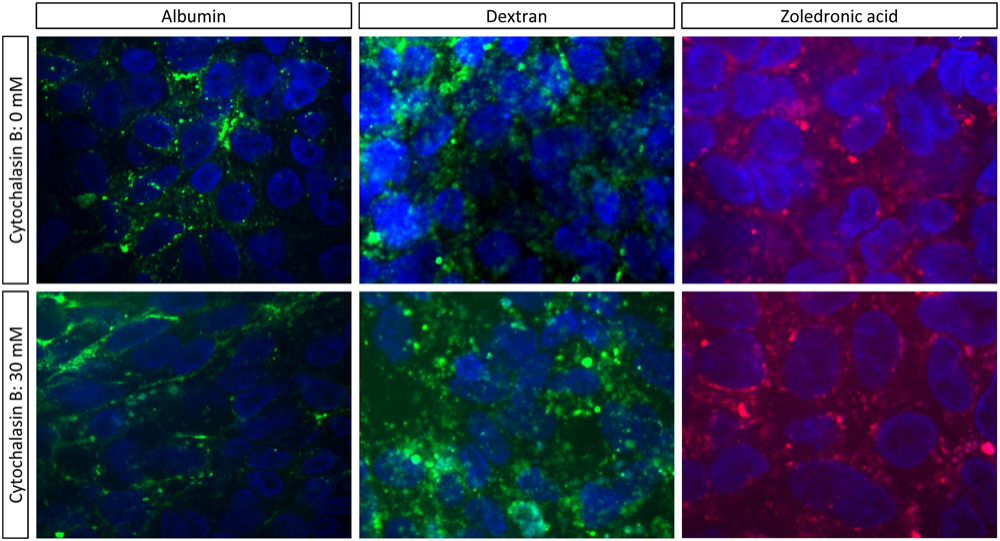
Confluent monolayers of human tubular kidney cells were incubated for 45 min with 0 or 30μM cytochalasin B, after which the monolayers were incubated 1 hour with either FITC-labeled dextran, or FITC-labeled albumin or AF647-labeled zoledronic acid, at 37°C, formalin fixed and counterstained with Hoechst.

Moreover results obtained after incubating cells with cytochalasin B further confirms a common uptake pathway for zoledronic acid and dextran. Whilst cytochalasin B resulted in a clear inhibition of albumin uptake no effect was seen for dextran/zoledronic acid (Fig. 6). Following cytochalasin B incubation the green labeled albumin is located along the cellular membrane and not as vesicles within the cells. These results indicate that dextran and zoledronic acid are taken up by a common, uptake route, i.e. fluid phase endocytosis while albumin is taken up by a different (receptor-mediated) endocytotic process, inhibited by cytochalasin B.

### Quantification of cellular uptake/intracellular levels of zoledronic acid

**Fig 7:**
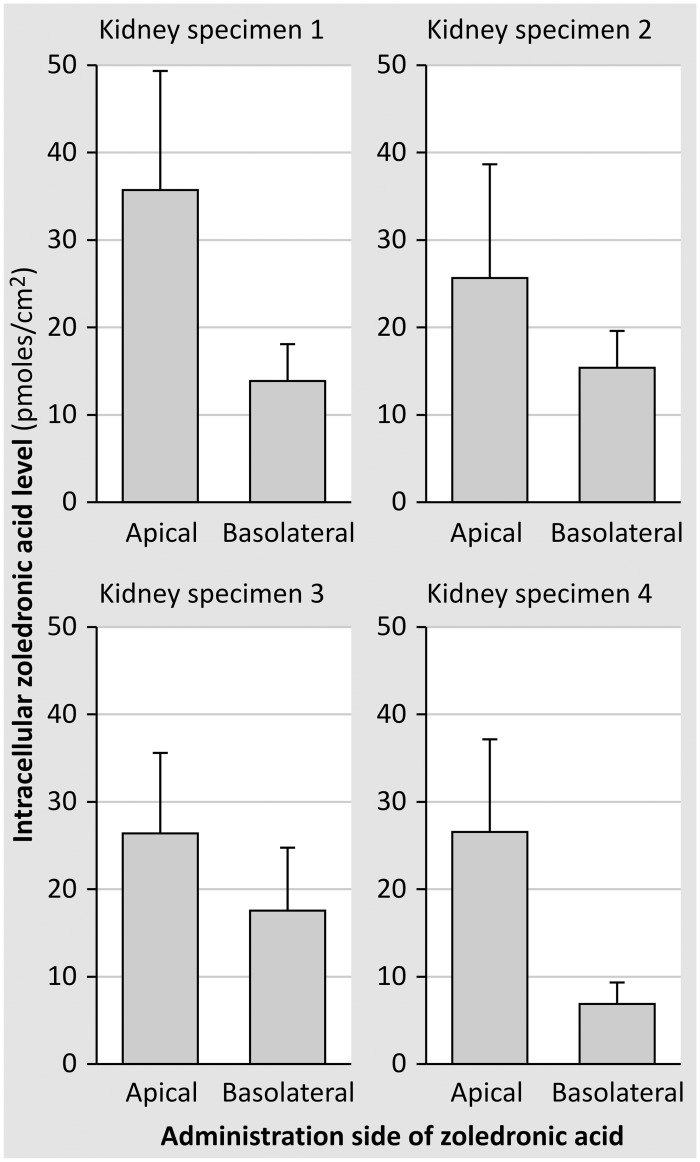
Intracellular accumulation of zoledronic acid was measured in confluent monolayers of primary human tubular cells using the steady state set-up. This figure shows the results of 4 experiments on monolayers originating from 4 different kidney specimens (for each experiment at least 5 monolayers/condition). Intracellular concentration was calculated as pmoles/cm^2^

Fig. 7 shows the intracellular levels of ^14^C-labeled-zoledronic acid in primary cell cultures derived from 4 different kidney specimens. Again, these experiments indicate that zoledronic acid is taken up from both the apical and the basolateral side and furthermore show that intracellular levels become significantly (p<0.0005) higher when administered at the apical side (28.7±11.8 pmoles/cm^2^) compared to the basolateral side (13.2±5.9 pmoles/cm^2^). In comparison, the intracellular levels of mannitol were only 3.3 and 4.0±1.0 pmoles/cm^2^, when administered to either the apical or basolateral side, respectively.

### Effect of organic anion transporter substrates and pamidronate on intracellular levels of zoledronic acid

**Fig 8:**
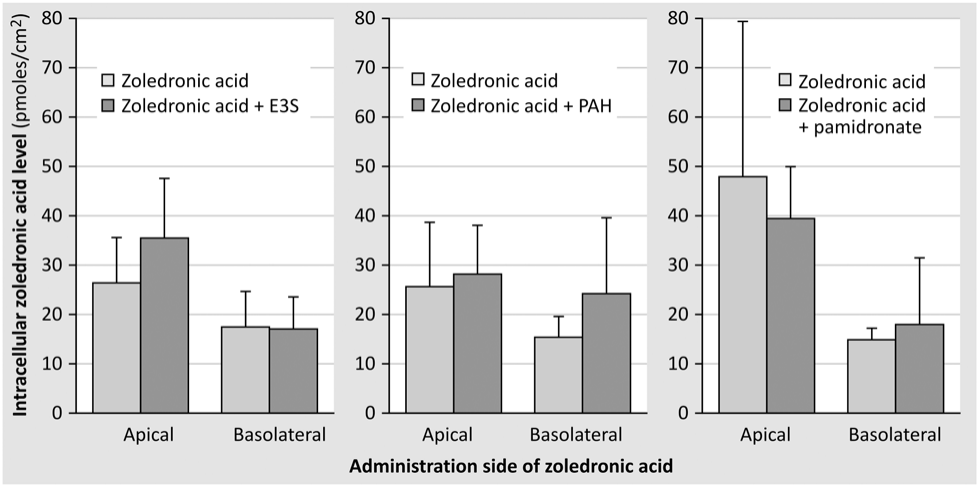
Intracellular concentration of zoledronic acid was measured in confluent monolayers of primary human tubular cells using the steady state set-up, in the pre- or absence of E3S/PAH/pamidronate (25μM). Each figure represents the results of one representative experiment on cells originating from one kidney (at least 5 monolayers/condition). Intracellular zoledronic acid concentration was calculated as pmoles/cm^2^.

An excess of organic anion transporter substrates PAH/E-3S was used in order to investigate whether zoledronic acid uptake into renal tubular cells is mediated by organic anion transporters. An excess of pamidronate, another N-containing bisphosphonate, was used in order to check for a common uptake pathway of both bisphosphonate molecules. Since intracellular levels of zoledronic acid did not change in the presence of an excess of the organic anion transporter substrates PAH and E-3S (Fig. 8), neither did the administration of pamidronate exert any effect on the intracellular levels of zoledronic acid (Fig. 8) no arguments were obtained for zoledronic acid uptake by organic anion transporters nor for a common uptake route of both bisphosphonates.

### Transepithelial transport of zoledronic acid

**Fig 9:**
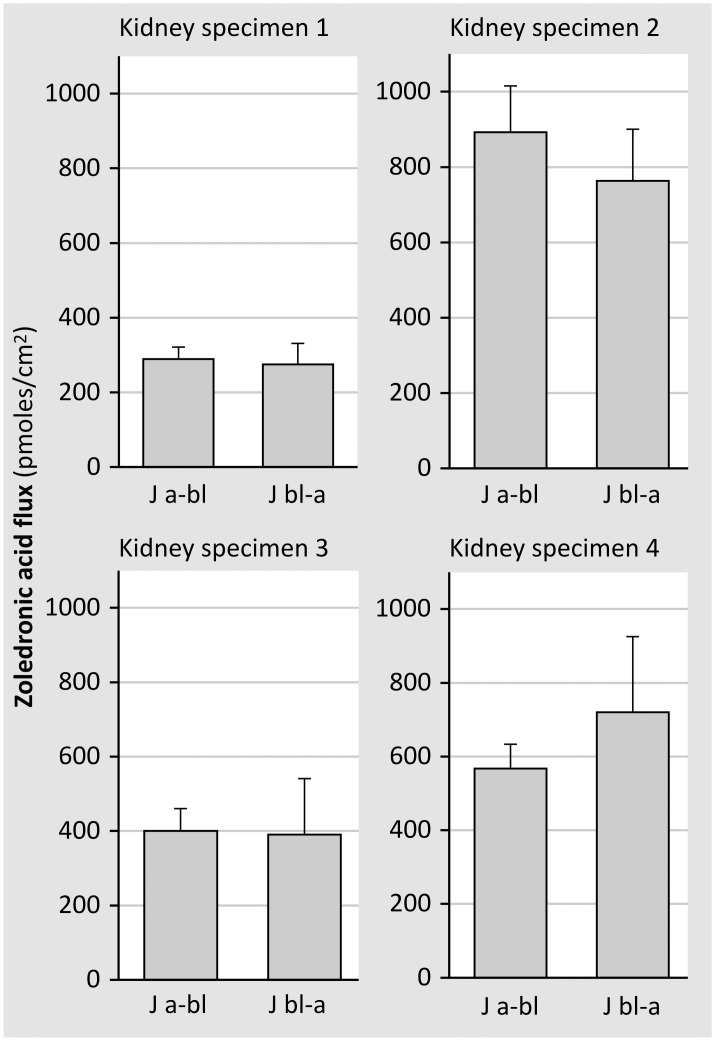
Ja-bl and Jbl-a fluxes of zoledronic acid were measured in confluent monolayers of primary human tubular cells using the steady state set-up. This figure shows the results of 4 experiments on monolayers originating from 4 different kidney specimens (for each experiment at least 5 monolayers/condition). Transepithelial fluxes in either direction did not significantly differ from each other.

**Fig 10:**
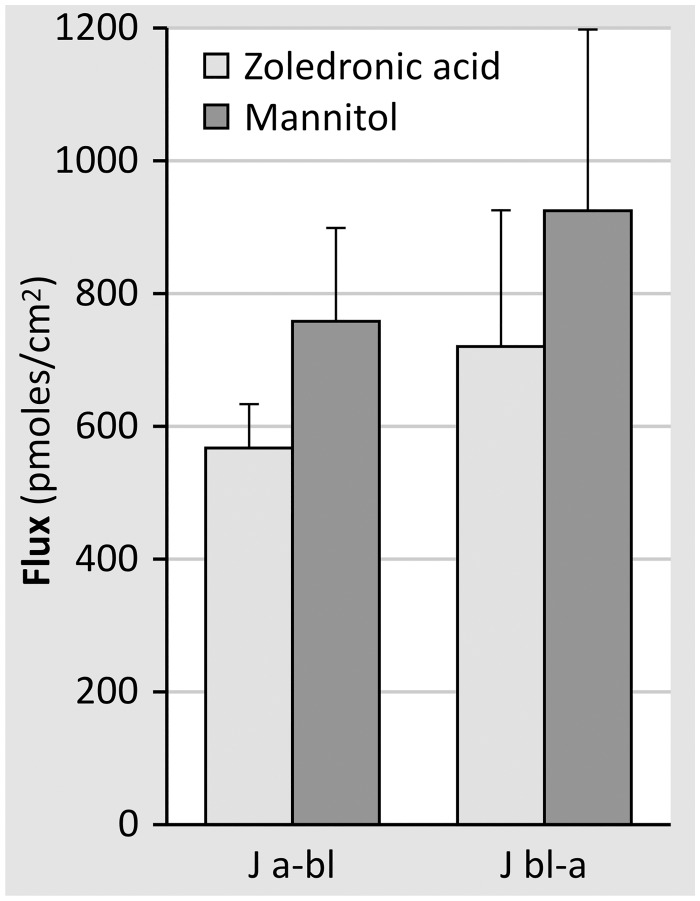
Ja-bl and Jbl-a fluxes of zoledronic acid and mannitol were measured in confluent monolayers of primary human tubular cells using the steady state set-up. This figure represents the results of one representative experiment on cells originating from one kidney (at least 5 monolayers/condition).

We next investigated whether cellular accumulation of zoledronic acid went along with net transcellular zoledronic acid fluxes. Fluxes directed from either the apical to the basolateral side (Ja-bl) or the basolateral to the apical side (Jbl-a) were measured in monolayers originating from 4 different kidney specimens. Results showed fluxes in both directions to be almost equal to each other (Fig. 9), suggesting that zoledronic acid transport takes place by the paracellular route. This was further confirmed by the finding that the transepithelial fluxes of zoledronic acid were not higher than those of mannitol (Fig. 10).

### Concentration dependence of tubular handling of zoledronic acid

**Fig 11:**
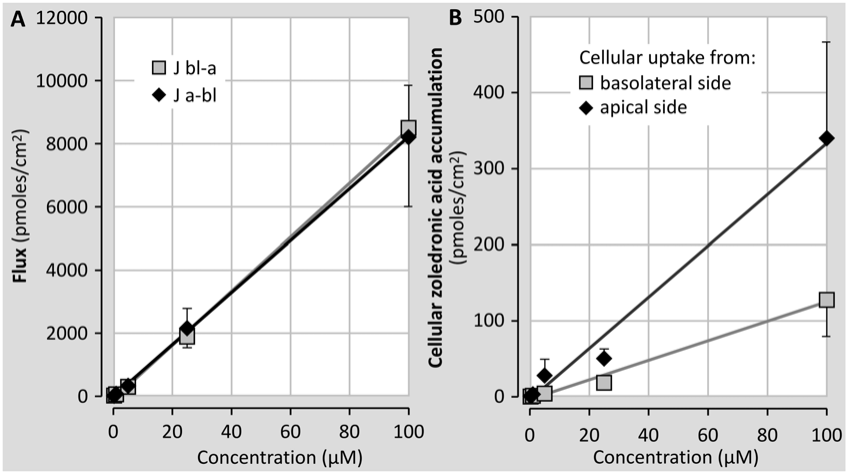
Administered concentration-dependence (0.25–100 μM) of zoledronic acid fluxes (transport). **(A)** and intracellular zoledronic acid concentration **(B)**. Zoledronic acid transport and intracellular accumulation was measured in confluent monolayers of primary human tubular cells using the steady state set-up. This figure represents the results of one representative experiment on cells originating from one kidney (at least 5 monolayers/condition).

To check the possible effect of the zoledronic acid concentration on its transport and intracellular accumulation cell cultures were incubated with different concentrations of the compound ranging from 0.25 to 100 μM (Fig. 11). Results in Fig. 10A show that fluxes from the apical to the basolateral side (Ja-bl) and from the basolateral to the apical side (Jbl-a) did not differ from each other over the whole concentration range. Moreover, the finding that no plateau is reached, provides an additional indication that the transport exclusively takes place by the paracellular way.

The effect of the zoledronic acid concentration on its intracellular level is shown in Fig. 11B. Again, the intracellular accumulation does not reach a plateau within the wide concentration range investigated. The latter finding is indicative for the fact that cellular accumulation of zoledronic acid is not regulated by membraneous protein transport molecules, as these would reasonably become saturated at the high concentrations applied in the present experiment.

## Discussion

Zoledronic acid is a well-known therapeutic agent used to decrease bone resorption in patients with osteoporosis (Reid *et el*., 2002). In addition the compound is also used at relatively higher doses in patients with multiple myeloma, lung, breast, and prostate cancer [1–3]. Due to its high affinity to hydroxyapatite, the majority of the administered zoledronic acid is presumed to accumulate in the bone. The zoledronic acid that does not bind to the bone is excreted, unmetabolized by the kidney, predominantly within the first hours after administration [4;10]. Since the administration of high doses of zoledronic acid may be associated with acute tubular necrosis in a number of patients [11], zoledronic acid uptake/accumulation by tubular cells probably occurs. Indeed, fluorescently labeled risedronate, which structurally is similar to zoledronic acid, has been detected in bone but also in renal tissue after administration to mice [14].

Using the previously described primary human tubular cell culture set-up, important aspects of tubular zoledronic acid handling were investigated. A zoledronic acid concentration range of 0.0, 0.25, 1.0, 5.0, 25 and 100 μM (corresponding to 0, 67.5, 270, 1350, 6750 and 27.000 ng/ml) was used. Since the serum zoledronic acid Cmax levels in patients after a 15 min intravenous infusion of 4mg zoledronic acid are around 300ng/ml [10], the lowest concentrations used in our experimental set-up thus perfectly correspond to serum levels in zoledronic acid treated patients. By using the higher zoledronic concentrations (25 and 100μM) we were able to demonstrate that zoledronic acid uptake/transport was not satiable.

It could clearly be shown that zoledronic acid uptake by tubular cells takes place by fluid phase endocytosis. This is in line with the observation of Roelofs et al. showing that cellular uptake of zoledronic acid and its analogue risedronate preferentially takes place in cell types with a high fluid phase endocytotic capacity such as osteoclasts and monocytes/macrophages [13;14].

To rule out a possible effect of the large chromophore on the uptake of labeled zoledronic acid, two different fluorescent labels were chosen, which showed consistent results thus indicating that the uptake of zoledronic acid by the tubular cells was determined predominantly by the parent drug rather than the conjugated fluorescent dyes. This is further supported by the fact that radiolabeled zoledronic acid also is taken up in the primary human tubular cells. Quantification of the uptake of radiolabeled zoledronic acid revealed a significantly higher (± 2-fold) uptake from the apical side as compared to the basolateral one. This finding is again in line with a fluid phase endocytotic uptake as it is known that the capacity of tubular cells to endocytose molecules directly correlates with the surface of the cellular membrane, which was reported to be twice as high at the apical compared to the basolateral side [36]. In this context it is worth to be mentioned that uptake of inulin by fluid phase endocytosis has also been reported to be two times higher at the apical side compared to the basolateral side [36]. Furthermore we also showed that the accumulation of zoledronic acid in tubular cells does not saturate at the high concentrations used in the present study. This further supports the uptake of zoledronic acid to occur by a process such as fluid phase endocytosis, which does not depend on the presence/availability of membraneous transporter proteins or receptors which would be required when uptake and transport would take place via specific (anion) transport mechanisms or receptor-mediated endocytosis.

Absence of zoledronic acid uptake by the proces of receptor- mediated endocytosis is evidenced by the finding that vesicles containing fluorescently labeled albumin clearly do not co-localize with zoledronic acid containing vesicles and by the fact that the uptake of zoledronic acid, in contrast to albumin, was not affected by incubating cells with cytochalasin B, a well-known inhibitor of receptor-mediated endocytosis. Moreover, the fact that on the one hand, receptor-mediated uptake of albumin is highly limited at the basolateral side of the tubular monolayers whilst on the other hand, uptake of zoledronic acid at this side is clearly visible and measurable again argues against receptor-mediated uptake of zoledronic acid.

The pathway of organic anion transport (OAT) is widely used by many compounds other than zoledronic acid. Using the same cell culture system we were able to show this pathway to be responsible for the uptake and secretion of e.g. rosuvastatin, a cholesterol lowering drug [18;35]. Absence of zoledronic acid uptake by this pathway is predicted by its structure: spacing of the anionic phosphate residues of zoledronic acid is not consistent with criteria for ligand interaction with any of the OATs, and is supported by our findings that neither PAH nor E-3S were able to inhibit accumulation of zoledronic acid in tubular cells.

The absence of net tubular transport (secretion) of zoledronic acid by the tubular cells is in accordance with the pharmacokinetic properties of zoledronic acid in humans, suggesting that tubular secretion of the compound does not take place. After infusion of zoledronic acid, its peak systemic concentration rapidly declines to <1% within 24 hours [4]. Renal clearance of zoledronic acid is positively correlated with but always smaller (on average 20%) than that of creatinine, a molecule known to be secreted to a certain extent [4]. Furthermore, measures of the degree of exposure such as AUC (area under the plasma concentration-time curve) and Cmax (peak plasma concentration) show dose-proportionality, suggesting that clearance of zoledronic acid does not rely on dose-dependent mechanisms, such as tubular secretion [4]. Moreover it has been shown for other N-containing bisphosphonates, including risedronate, ibandronate and pamidronate that renal excretion seemingly is mediated by glomerular filtration and not by tubular secretion [37–39].

The absence of a net measurable tubular transport/flux for zoledronic acid does not necessarily imply that this molecule is not secreted by the tubular cells. In osteoclasts zoledronic acid is released via transcytosis [40]. Lacave et al. described fluid phase endocytotic uptake of inulin by tubular cells and reported this molecule is taken up and secreted at both the basolateral and the apical side [36].

It is a limitation of this study that we did not investigate the mechnism by which the observed toxicity is induced. However, since bisphoshonate induced toxicity is a consequence of farnesyl diphosphate synthase inhibition in different cell-types (osteoclasts, monocytes), in our opinion there is no reason to believe that this should be not the case in renal tubular cells. Obviously, since also the uptake pathway for zoledronic acid in renal tubular cells seems to be identical to that in osteoclasts and monocytes.

In order to be able to fulfill its probable role as an inhibitor of farnesyl diphosphate synthase and in order to clarify its observed tubular toxicity, zoledronic acid must leave the endocytotic vesicles and be sequestered in the cytosol and/or mitochondria (in human cells the primary localizations of farnesyl diphosphate synthase [41]. The fact that cytosolic and/or mitochondrial localization of zoledronic acid could not be observed by using the fluorescently labeled compound is a limitation of this study. The quantification of radiolabeled zoledronic acid however, logically includes zoledronic acid located in all cellular organelles.

Viability of the tubular cells incubated with zoledronic acid was not acutely affected but decreased only on the longer term (48h). This fully agrees with data from a previous study investigating the inhibition of farnesyl diphosphate synthase enzyme and cytotoxicity of zoledronic acid in two renal cell lines [42]. The authors reported that farnesyl diphosphate synthase already was inhibited after 1h while cytotoxicity resulting from the decreased levels of prenylated proteins occurred after 48h. Furthermore, the fact that cytotoxity was only seen at higher concentrations (or after longer incubation periods) is in accordance with *in vivo* observations showing that renal damage declines when the dose is reduced [1] and with data from clinical studies demonstrating renal toxicity to be absent in postmenopausal women that receive zoledronic acid at relatively low annual dose [12]. These observations can be explained by the fact that protein prenylation must be reduced to below a critical level before cytotoxicity eventually occurs [42].

In conclusion, this manuscript presents evidence that zoledronic acid is internalized by renal tubular cells via the process of fluid phase endocytosis. The intracellular presence of zoledronic acid may induce tubular cytotoxicity provided its intracellular concentrations reach a high enough level. Our findings may contribute to a better understanding of the observed renal damage in particular patient populations receiving zoledronic acid at high doses.
